# Microbial degradation of Diquat by strain *Meyerozyma guilliermondii* Wyslmt: Identification of transformation products and clarification of degradation pathways

**DOI:** 10.3389/fmicb.2025.1652141

**Published:** 2025-08-06

**Authors:** Fangyuan Wang, Jinrong Jia, Wei Gu

**Affiliations:** Institute of Plant Protection, Heilongjiang Academy of Agricultural Sciences, Harbin, China

**Keywords:** Diquat, *Meyerozyma guilliermondii* Wyslmt, biodegradation, transformation product, UPLC-QTOF-MS

## Abstract

The continuous and extensive use of pesticides has negative impacts on the environment and human health. Microbial remediation is an eco-friendly and economically efficient technology, which is of great significance. In this investigation, the degradation of the herbicide Diquat by yeast Wyslmt was studied in medium under different conditions. The degradation rate of Diquat showed a pattern of first increasing and then decreasing with the increase of the inoculation amount of Wyslmt, temperature, pH and the initial concentration of Diquat. The biodegradation transformation products (BTPs) formed by the microbial degradation of Diquat in the culture medium solution were isolated and identified by ultra-high-performance liquid chromatography coupled with time-of-fight mass spectrometry (UPLC-QTOF-MS). Based on the mass spectrometry information, three main transformation products were determined. The calculation of components, the comparison of structural analogues, and the existing literature are all helpful for the determination of the structure. The main pathway of microbial degradation was C–C bond broken, hydroxylation and demethylation. These results lay the foundation for further environmental risk assessment and provide a reference for the bioremediation evaluation of bipyridine herbicides.

## 1 Introduction

Since the middle of the 20th century, the global production of traditional agricultural forms has been insufficient to meet the continuous growth of the Earth’s population, which has led to the widespread use of pesticides. Pesticides are difficult to completely degrade without intervention (Bi et al., 2023). The half-life of pesticide residues can last for several years (Concha and Manzano, 2023). Pesticides enter ecosystems through water and air (Bhattacharyya et al., 2022; Thompson et al., 2021; Mali et al., 2023; Gunier et al., 2018), concentrate along the food chain, and ultimately pose a potential serious threat to biodiversity and human health (Sulaiman et al., 2019). Unfortunately, pesticides have become an indispensable part of modern agriculture. Therefore, it is necessary and urgent to develop pesticide residue degradation technology to provide guarantees for agricultural harvest and ecological civilization construction (Tarfeen et al., 2022). In this sense, compared with restricting the use of pesticides, a wiser choice is to formulate a robust pesticide residue degradation strategy, which can not only enhance environmental recovery but also maintain the sustainable development of the living environment (Bolan et al., 2023; Bokade et al., 2023).

Bioremediation is regarded as a highly promising method for removing chemical pollutants from water and soil environments (Chen et al., 2014; Yang et al., 2018). It is also considered an environmentally friendly, low-cost, and effective alternative chemical and physical technology that can currently be used to reduce various pollutants in the environment (Nicholls and Altieri, 2013). As pointed out by Guerrero Ramírez et al. (2023) microorganisms have a strong adaptability to constantly changing environments, such as inductions or mutations. Microorganisms utilize various metabolic pathways to break down these exogenous compounds, using them as sources of carbon, nitrogen, phosphorus, energy, and more. The microbial metabolism of pesticides ends in one of two situations: complete molecular biodegradation or mineralization. In such cases, most of the by-products are environmentally benign and suitable for re-entering the ecosystem (Matsumura, 1982; Dar et al., 2022; Maqbool et al., 2016). Under the action of microorganisms, pesticides may undergo reactions such as oxidation, dehalogenation, hydroxylation, demethylation, denitrification, desulfurization, decarboxylation, ammonification and hydrolysis (Karigar and Rao, 2011). An increasing number of microorganisms have been found capable of degrading pesticide residues. However, although fungi exhibit greater metabolic diversity than bacteria, they have been less extensively studied (Guerrero Ramírez et al., 2023).

Diquat is a non-selective contact bipyridine herbicide used to control the growth of broadleaf and grassy weeds in aquatic and terrestrial non-crop areas. It is one of the most commonly used herbicides worldwide. Diquat is physically more suitable for agricultural use than many other herbicides due to its crystallinity (making treatment easier), high water solubility of 700 g/L at 20°C (better efficacy), low vapor pressure (minimal smoke), fast working (once photosynthesis begins), and high binding potential (soil binding leading to inactivation and immobilization) (Shibin et al., 2015; Wang and Tao, 2025). Therefore, Diquat is highly likely to cause environmental pollution. At present, the microbial repair of the Diquat and its degradation products and mechanisms have not attracted people’s attention. In the early stage, we screened out a strain of *Meyerozyma guilliermondii* Wyslmt which can efficiently degrade the Diquat (Wang et al., 2022). The purpose of this study is: (1) to analyze the influence of different medium environmental conditions of Wyslmt on the degradation kinetics of Diquat; (2) determine the chemical structure of microbial degradation products in medium by the UPLC-QTOF-MS technique; and (3) to provide a reference for evaluating the bioremediation of bipyridine herbicides.

## 2 Materials and methods

### 2.1 Yeast strain *Meyerozyma guilliermondii* Wyslmt

*Meyerozyma guilliermondii* Wyslmt showed high degradation rate of Diquat (100 mg/L) (7d, 42.51%) in our previous study (Wang et al., 2022) was further analyzed at the transcriptomic level in order to identify the genes involved in the biodegradation of Diquat of strain Wyslmt (deposited in GenBank under the name “Wyslmt” with the accession number MZ520358; Bioproject accession: PRJNA809846).

### 2.2 Chemicals and reagents

Diquat (99.9%) was purchased from the Putian Genesis Biotechnology Co., Ltd. (Beijing, China). The other chemical substances used in this study were analytical grade chemical substances, and the chemical substances used for high performance liquid chromatography (HPLC) analysis were HPLC grade chemical substances. Potato dextrose broth (PDB) medium was consisted of 200.0 g of potatoes and 20.0 g of glucose per liter. Diquat was added to the medium at appropriate concentrations to yield Diquat-supplemented PDB. Medium was sterilized via autoclaving for 30 min at 121°C.

### 2.3 Biodegradation experiments

Wyslmt strain yeast were cultured to the logarithmic growth phase (OD_600_ = 0.6) to prepare a stock solution from which a 1% inoculum was added into 100 mL of PDB containing a certain concentration of Diquat (Wang et al., 2022). Different temperatures (20°C, 24°C, 28°C, 32°C, 36°C, and 40°C), pH values (4.0, 5.0, 6.0, 7.0, 8.0, 9.0, and 10.0), inoculation amounts of strain Wyslmt (0.1, 0.5, 1, 3, 5, 7, 9, and 11%) and different initial concentrations of Diquat (1 mg/L, 10 mg/L, 50 mg/L, 100 mg/L, 200 mg/L, and 300 mg/L) were set. Under the conditions of 28°C and continuous stirring (180 revolutions per minute), the concentration of Diquat and the growth of yeast Wyslmt on the 7th day of culture were determined. The absorbance of the culture supernatant was determined at 600 nm using the TU-1901 spectrophotometer (Beijing Purkinje General Instrument Co., Ltd., China) to monitor the growth of yeast. The concentration of Diquat was determined by HPLC. Each experiment was conducted at least three times, the control solution was prepared without any initial inoculation, and the results, where available, were expressed as the average value of the ±95% confidence intervals.

### 2.4 Calculation of Diquat degradation rate

The initial and final concentrations of Diquat were determined by HPLC, and the removal rate of Diquat was calculated. The percentage of Diquat degradation was calculated as follows:


$$X=(C_{\mathrm{CK}}-C_{X})/C_{\mathrm{CK}}\times 100\%$$

where X represents the degradation rate of Diquat, C_CK_ is the initial concentration of Diquat (mg/L), and C_X_ is the final concentration of Diquat (mg/L).

### 2.5 Analytical methods

The concentration of Diquat was determined by HPLC (Ultimate 3,000). HPLC adopted a variable-wavelength ultraviolet detector of 308 nm, a reverse-phase C18 column (4.6 × 250 mm, 5 μm), a column temperature of 30°C, and a flow rate of 1.0 mL/min (acetonitrile/water = 40/60, v/v). All injection volumes were 10 μL (Wang et al., 2022). Diquat and its BTPs were separated using the UPLC system (Acquity I Class Waters Corp, Milford, MA, United States). The column temperature was 35°C and the injection volume was 10 μL. Diquat and its BTPs were identified using a QTOF mass spectrometer (X500, AB Science, SECIEX, CA, USA) under the following flow conditions: The electrospray ionization source (ESI) was the ion source, in the positive ion mode, with a mass scanning range of m/z 50–1,000, a spray voltage of 5,500 V, an air curtain flow rate of 30 L/min, a declustering voltage (DP) of 80 V, and a temperature of 400°C. Data were collected using the TOF-MS-IDA-MS/MS method. TOF/MS first-level pre-scanning and triggering. The secondary scan adopted TOF/MS/MS with ion accumulation times of 200 and 100 ms respectively, the CE collision energy was 35 eV, and the collision energy spread (CES) was ±15 eV (Wang and Tao, 2025).

In this study, the limits of detection (LOD) and limits of quantification (LOQ) values for Diquat were 0.02 mg/L and 0.05 mg/L for PDB medium, respectively. The recovery rate of the spiked samples at the three added levels of Diquat (2 mg/L, 5 mg/L, 10 mg/L) was 96.3–98.6%.

## 3 Results and discussion

### 3.1 The degradation effect of the strain Wyslmt on Diquat under different culture conditions

**Figure 1:**
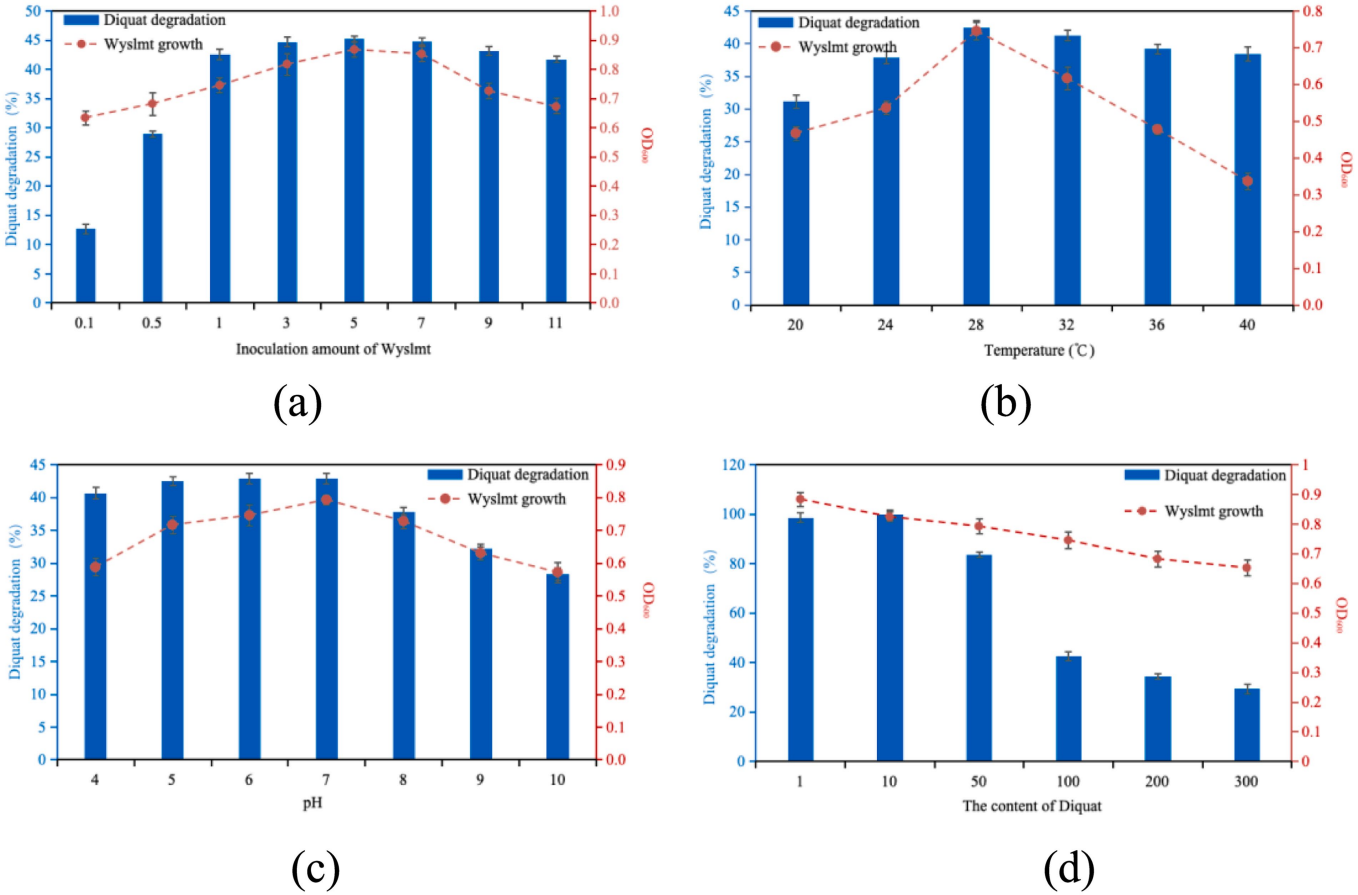
The effect of inoculation amount of Wyslmt **(a)**, temperature **(b)**, pH **(c)**, and Diquat content **(d)** on the Biodegradation of growth of the Wyslmt strain and associated Diquat degradation. Error bars represent the standard deviation for three replicate samples. The error bar represents standard error (SD) of the mean of the three replicates. Values are expressed as the means ± standard error (SE) of three replicates.

Research results indicate that a higher concentration of microbial populations leads to a higher degradation efficiency of pesticides (Zabaloy and Gómez, 2014; Vanitha et al., 2023; Serbent et al., 2019). This is similar to our research results. Different inoculation amounts of strain Wyslmt were added to the PDB medium with a concentration of 100 mg/L of Diquat, and the degradation rate was determined after shaking culture at 28°C for 7 days. The results were shown in Figure 1a. When the inoculation amount is 1–11%, the degradation rate was 41.70–45.28%. The degradation rate of Diquat by strain Wyslmt changed little. The degradation rate varies greatly when the inoculation amount was between 0.1 and 1%. Furthermore, the research found that when the inoculation amount of Wyslmt exceeded 5%, both the growth of Wyslmt and the degradation rate of Diquat began to decline. This might be because Wyslmt degrades Diquat through co-metabolism. An excessively high inoculation amount leads to rapid consumption of nutrients, thereby affecting the degradation efficiency of Diquat. Comprehensively considering multiple factors such as the need for horizontal comparison of different treatments in the experiment, as well as ensuring the accuracy of the experiment so that the inoculation amount cannot be too large. Therefore, in the degradation test of Diquat, the inoculation dose of the selected strain Wyslmt was 1%.

The 1% strain Wyslmt was added to the PDB medium with a concentration of 100 mg/L of Diquat. The degradation rates after 7 days of culture under different temperature conditions were shown in Figure 1b. Different temperatures had a significant impact on the degradation rate of strain Wyslmt. When the temperature was 20°C, the degradation rate of the strain Wyslmt was 31.13%. With the increase of temperature, the degradation rate gradually increased and reached the maximum degradation rate of 42.51% at 28°C. Subsequently, the degradation rate of the strain Wyslmt decreased with the increase of temperature. This is because temperature has a significant impact on the degradation process because it affects the function and activity of enzymes (Jaiswal et al., 2017). The rate of enzymatic catalytic reactions directly affects the rate of biodegradation (Gangola et al., 2022). Therefore, both excessively high and low temperatures can inhibit the degradation ability of the strain Wyslmt to Diquat.

Each type of microorganism has its optimal pH range for growth and maximum function. When the pH value is lower or higher than the optimal pH range, their metabolic activity will also decrease or increase (Gangola et al., 2022). Cycoń et al. (2009) observed that pH value has a significant impact on the degradation of pesticides. The degradation rate of the culture at 28°C under different pH values for 7 days was shown in Figure 1c. When the pH value was 4–7, the change of degradation rate was relatively gentle. The degradation rate was within the range of 40.67–42.85%. Then, with the increase of pH value, the degradation rate gradually decreased. Xia et al. (2017) found that when the pH was lower than 6.0 and higher than 9.0, cell division and the degradation of 2, 4-D were significantly hindered, indicating that highly alkaline conditions might have a negative impact on the metabolism of 2, 4-D. This also indirectly indicates that the strain Wyslmt can be used as a better bioremediation material, as long as it is not under non-extreme pH conditions.

The 1% strain Wyslmt was added to the PDB medium containing different concentrations of Diquat and cultured at 28°C for 7 days. The degradation rates were shown in Figure 1d. The degradation ability of this strain in 1 mg/L, 10 mg/L and 50 mg/L Diquat medium was significantly higher than that in 100 mg/L, 200 mg/L and 300 mg/L Diquat content medium. Among them, the degradation rate was the highest in the medium with a Diquat concentration of 10 mg/L. It was 99.74%. Then, with the increase of the concentration of Diquat, the degradation rate of the strain in the culture medium gradually decreased. When the concentration of Diquat increased to 300 mg/L, the degradation rate was 29.41%. *Lipomyces starkeyi* Lod and Rij completely removed paraquat (27 mg/L) from the culture medium within 3 days. However, when the concentration of paraquat doubled (54 mg/L), the biomass and degradation of paraquat significantly decreased to below 10% (Alexander, 1999). This is consistent with our discovery.

### 3.2 BTPs analysis

**Figure 2:**
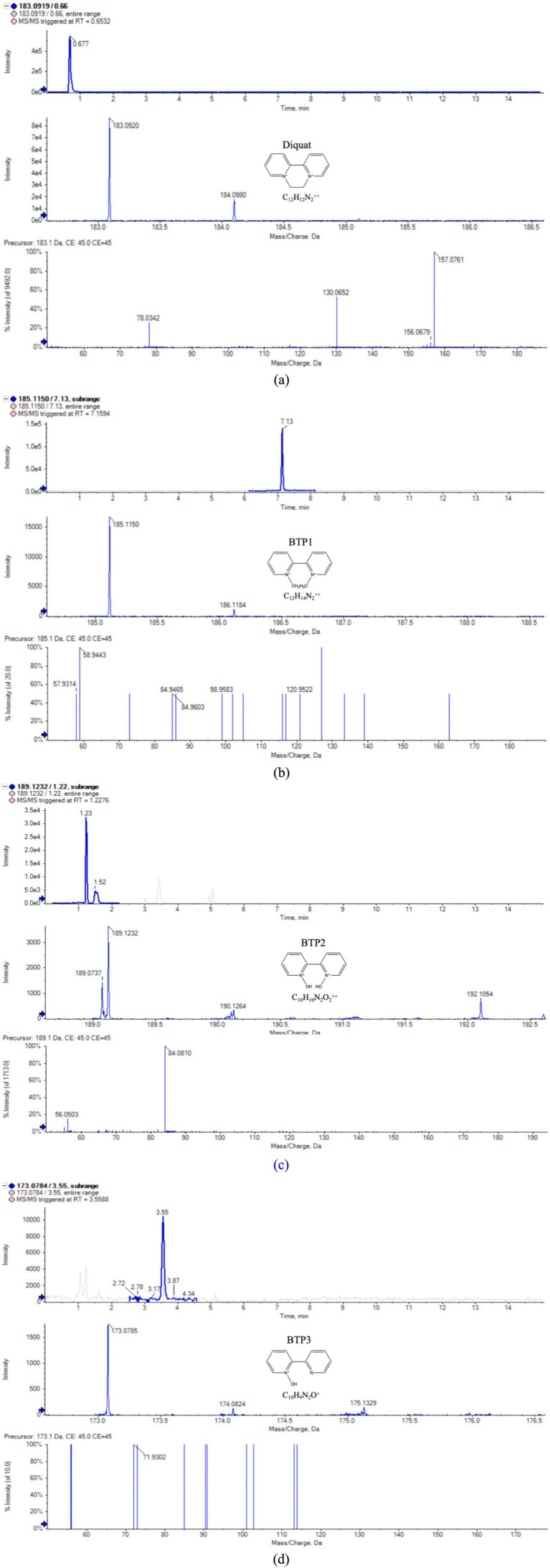
Extraction of ion chromatogram, MS and MS/MS spectra of Diquat and its BTPs **(a–d)** are Diquat, BTP 1–BTP 3, respectively.

Some studies have proposed that when there was a lack of the literature and standards information for confirmation, as well as there was a short of other structures can match the test information, diagnostic MS/MS fragments, information about the parent compound, ionization behavior, and the test background can been the basis of compound identification (Li and Hu, 2024). Therefore, in this study, UPLC-QTOF-MS was adopted to separate and analyze Diquat and BTPs. Most microorganisms only carry out one or two steps in the degradation process, leaving behind potentially toxic intermediate metabolites (Bi et al., 2023). We only found that three BTPs were produced after Diquat was cultured in the dark in PDB medium. Figure 2 and Table 1, respectively, showed the typical chromatogram and the mass spectrometry information of Diquat and its BTPs, as well as the cationic or molecular formula, retention time, cationic or molecular mass, and product ions.

**Table 1:** Mass spectra data of the identified BTPs using UPLC-QTOF-MS.

Compound	Molecular or cationic formula	RT (min)	Molecular or cationic mass (Da)	Product ion
Diquat	C_12_H_12_N_2_^++^	0.66	184	157 [M^+•^-H^•^-C_2_H_2_]^+^ 130 [M^+•^-H^•^-C_2_H_2_-HCN]^+^ 78 [M^+•^-H^•^-C_2_H_2_-HCN-C_4_H_4_]^+^
BTP 1	C_12_H_14_N_2_^++^	7.13	186	121 [M^+•^-H^•^-C_5_H_4_]^+^ 85 [M^+•^-H^•^-C_5_H_4_-3C]^+^ 59 [M^+•^-H^•^-C_5_H_4_-3C-C_2_H_2_]^+^
BTP 2	C_10_H_10_N_2_O_2_^++^	1.22	190	84 [M^+•^-H^•^-C_4_H_4_-C_4_H_4_-H]^+^ 56 [M^+•^-H^•^-C_4_H_4_-C_4_H_4_-H-C-O]^+^
BTP 3	C_10_H_9_N_2_O^+^	3.55	173	71 [M^+^-C_6_H_6_-2C]^+^

According to the mass spectrometry data, we speculated the cationic formula of BTP 1 was C_12_H_14_N_2_^++^, the peak appeared at 7.13 min, the ion [M^+•^-H^•^] was at m/z 185. The product ions of BTP 1 was at m/z 121 [M^+•^-H^•^-C_5_H_4_]^+^, 85 [M^+•^-H^•^-C_5_H_4_-3C]^+^ and 59 [M^+•^-H^•^-C_5_H_4_-3C-C_2_H_2_]^+^. The cationic weight of BTP 1 was 2 Da larger than Diquat, based on its fragment ions and possible cationic formulas, it was inferred that it was the result of the cleavage of the [CH_2_-CH_2_] bond on the piperazine ring of Diquat, forming two [CH_3_]s, respectively. It can be found from many studies that ring-opening was a common way for microorganisms to degrade pesticides (Malla et al., 2023; Funderburk and Bozarth, 1967; Xu et al., 2022; Mishra et al., 2020; Magnoli et al., 2020; Li et al., 2020). Slade (1965) proposed that the degradation of paraquat was initiated by opening the pyridine ring between the N atom and the adjacent C atom. The formation of BTP 1 was due to the cleavage of the C-C bond of the piperazine ring. Therefore, we considered this to be the first step for Wyslmt to degrade the Diquat.

Based on the mass spectrometry information of BTP 2, we believed that C_10_H_10_N_2_O_2_^++^ might be its chemical formula, its cationic weight was 190, its peak occurred at 1.22 min, and its main product ions were at m/z 84 [M^+•^-H^•^-C_4_H_4_-C_4_H_4_-H]^+^ and 56 [M^+•^-H^•^-C_4_H_4_-C_4_H_4_-H-C-O]^+^. Hydroxylation is the most likely reaction to occur during the degradation of pesticides, mainly due to the presence of oxygen free radicals in the environment that can attack the weak chemical bonds of pesticides. At the same time, microorganisms will also carry out oxidation, hydroxylation, demethylation and other reactions according to the different chemical components of pesticides (Karigar and Rao, 2011). Many research results proved that hydroxylation was one of the more common and important reactions in the degradation process of pesticides (Magnoli et al., 2020; Huang et al., 2021). The cationic weight of BTP 2 was 4 Da greater than that of BTP 1. So BTP 2 was regarded as the result of the complete hydroxylation of CH_3_ in BTP 1.

Paraquat, also a bipyridine herbicide, undergoes the first degradation reaction during microbial degradation, which was demethylation, forming Monoquat (Huang et al., 2019). Demethylation is also one of the common reactions in the microbial degradation process of other pesticides. For example, during the process of *T. versicolor* degrading Malathion (Hu et al., 2022), during the process of bacteria degrading Dicamba, and so on (Li et al., 2020). The mass spectrum peak of BTP 3 appeared at 3.55 min, with its ion located at m/z 173, its main product ion were situated at m/z 71 [M^+^-C_6_H_6_-2C]^+^. Based on the above information, the molecular formula we inferred was C_10_H_9_N_2_O^+^. According to its fragment ions and possible cationic formulas, it was inferred BTP 3 was the result of demethylation and then hydroxylation of BTP 1.

### 3.3 Microbial degradation pathway of Diquat

**Figure 3:**
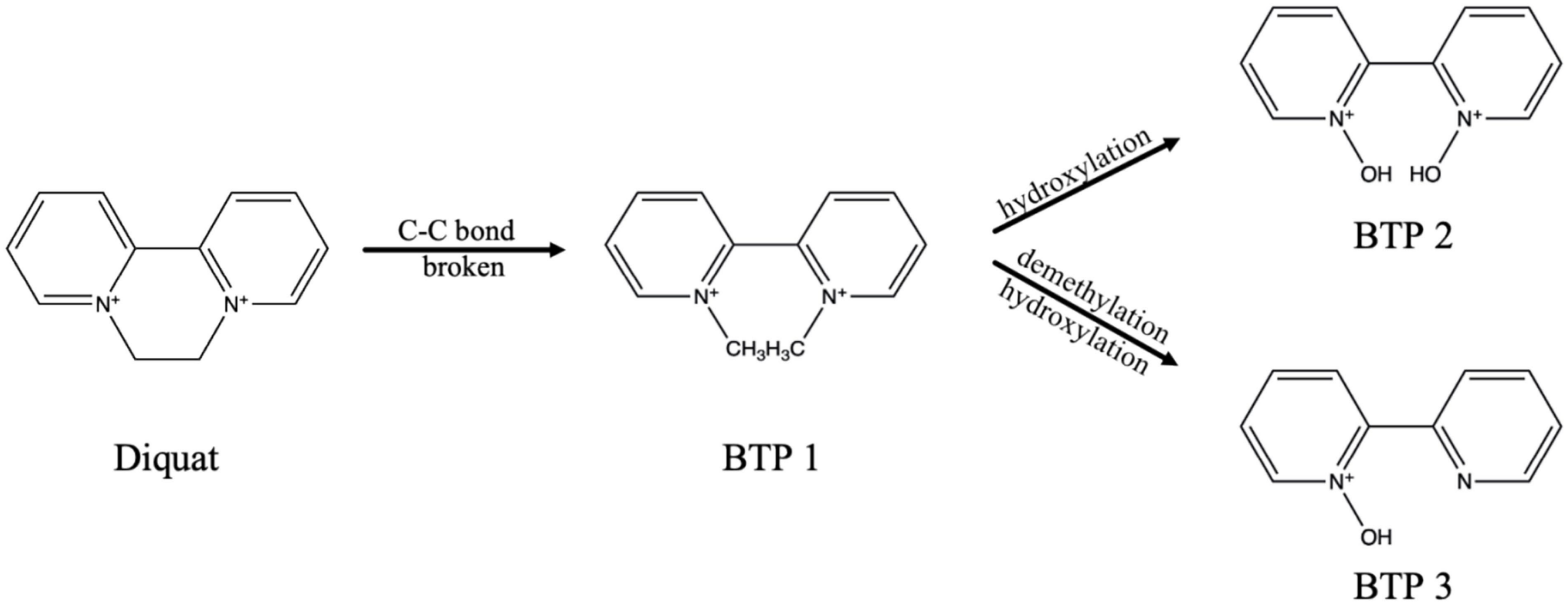
Proposed microbial degradation pathways of Diquat in PDB medium.

Based on the microbial degradation products, the Diquat microbial degradation pathways were inferred, indicating that Diquat may have one microbial degradation pathways. Figure 3 listed three possible degradation pathways: (1) C-C bond broken; (2) hydroxylation; (3) demethylation.

## 4 Conclusion

The degradation of the herbicide Diquat by yeast Wyslmt was studied in PDB medium under different conditions. The transformation products were identified by UPLC-QTOF-MS/MS. The degradation rate of Diquat showed a pattern of first increasing and then decreasing with the increase of the inoculation amount of Wyslmt, temperature, pH and the initial concentration of Diquat. These products were separated and identified by using UPLC-QTOF-MS/MS, the main biodegradation pathways of Diquat were C-C bond broken, hydroxylation and demethylation. The results provide a reference for the bioremediation evaluation of bipyridine herbicides.

## Data Availability

The original contributions presented in the study are included in the article/supplementary material, further inquiries can be directed to the corresponding authors.
